# Systematic review and meta-analysis of the participation rates in clinical trials evaluating non-pharmacological interventions for psychosis

**DOI:** 10.1186/1745-6215-16-S2-P169

**Published:** 2015-11-16

**Authors:** Paulina Szymczynska, Sophie Walsh, Stefan Priebe

**Affiliations:** 1 Queen Mary University of London, London, UK

## 

Poor retention of participants in randomised controlled trials (RCTs) is a commonly recognised issue. Attrition rates in studies across different disciplines have been reported to vary from 5% to 70%. There is no systematic evidence reporting attrition rates specifically in trials involving people with severe and fluctuating mental health conditions.

To purpose of this review was to synthesise the reported retention rates in RCTs evaluating non-pharmacological interventions for psychosis and identify study characteristics associated with high dropout.

Five key electronic databases and key journals were systematically searched to identify studies published between 1996 and 2015. The search included large scale RCTs (n≥100 at baseline) including adults with psychosis receiving non-pharmacological interventions. Data extraction included participant flow information reported in the Consolidated Standards of Reporting Trials (CONSORT) statement or flow diagram. Random effects logistic regression analysis was conducted to examine effects of key study characteristics on participation rates.

62 papers were included in the review. This paper will discuss the participation rates reported in RCTs, the quality of reporting in publications, and the study characteristics that predict participation rates.

